# Clinical diagnosis and treatment of common respiratory tract infections in relation to microbiological profiles in rural health facilities in China: implications for antibiotic stewardship

**DOI:** 10.1186/s12875-021-01448-2

**Published:** 2021-05-06

**Authors:** Xingrong Shen, Jilu Shen, Yaping Pan, Jing Cheng, Jing Chai, Karen Bowker, Alasdair MacGowan, Isabel Oliver, Helen Lambert, Debing Wang

**Affiliations:** 1 School, of Public Health, Anhui Medical University, Hefei, Anhui China; 2 School of Health Service Management, Anhui Medical University, Hefei, Anhui China; 3 Department of Clinical Laboratory, the Fourth Affiliated Hospital of Anhui Medical University, Hefei, Anhui China; 4 Department of Clinical Laboratory, the First Affiliated Hospital of Anhui Medical University, Hefei, Anhui China; 5 Infection Sciences, Severn Pathology, North Bristol NHS Trust, Pathology Building, Southmead Hospital, Westbury-On-Trym, Bristol, BS10 5NB UK; 6 Field Service, National Infection Service, Public Health England, 3rd floor, 2 Rivergate, Bristol, BS1 6EH UK; 7 Bristol Medical School, University of Bristol, Bristol, UK

**Keywords:** Antibiotic, Respiratory tract infection, Primary care, Diagnosis, Microbiological, China

## Abstract

**Background:**

This paper tries to describe prevalence and patterns of antibiotics prescription and bacteria detection and sensitivity to antibiotics in rural China and implications for future antibiotic stewardship.

**Methods:**

The study was implemented in one village clinic and one township health center in each of four rural residential areas in Anhui Province, China. It used mixed-methods comprising non-participative observations, exit-survey and microbiological study. Observations were conducted to record clinical diagnosis and antibiotic prescription. Semi-structured questionnaire survey was used to collect patient’s sociodemographic information and symptoms. Sputum and throat swabs were collected for bacterial culture and susceptibility testing.

**Results:**

A total of 1068 (51.0% male vs 49.0% female) patients completed the study with diagnosis of respiratory tract infection (326,30.5%), bronchitis/tracheitis (249,23.3%), pharyngitis (119,11.1%) and others (374, 35.0%). They provided 683 sputum and 385 throat swab specimens. Antibiotics were prescribed for 88% of the RTI patients. Of all the specimens tested, 329 (31%) were isolated with bacteria. The most frequently detected bacteria were *K. pneumonia* (24% in all specimens), *H. influenza* (16%), *H. parainfluenzae* (15%), *P. aeruginosa* (6%)*, S.aureus* (5%), *M. catarrhalis* (3%) and *S. pneumoniae* (2%).

**Conclusions:**

The study establishes the feasibility of conducting microbiological testing outside Tier 2 and 3 hospitals in rural China. It reveals that prescription of antibiotics, especially broad-spectrum and combined antibiotics, is still very common and there is a clear need for stewardship programs aimed at both reducing the number of prescriptions and promoting single and narrow-spectrum antibiotics.

**Supplementary Information:**

The online version contains supplementary material available at 10.1186/s12875-021-01448-2.

## Background

Antimicrobial resistance (AMR) has become one of the biggest threats to global health [1, 2]. It leads to higher financial costs, prolonged hospital stays and increased patient mortality. Data from 76 countries including China show that global antibiotics consumption grew by more than 39% between 2000 and 2015 and China consumes the second largest amount of antibiotics in the world [3] with a prescription rate twice that recommended by the World Health Organization (WHO). Rural areas have higher antibiotic prescribing rates than urban areas [4]. According to a survey in rural areas of Shandong and Ningxia, 89% and 77% of the prescriptions contained antibiotics for patients clinically diagnosed as having upper respiratory tract infections (RTIs) [5]. Another survey of prescriptions from village clinics in middle-east China showed that the proportion of antibiotics prescribed for RTIs accounted for 87% [6]. The Chinese government has introduced a number of regulations to control antibiotic use in the last decade, but these have not played a significant role in rural areas [7].

The majority of people in China live in rural and township areas. Physicians in these areas mainly provide two kinds of services for residents, the treatment of common infectious diseases (mainly RTIs), and the management of chronic diseases. The excessive antibiotics prescriptions mostly occur in treating common RTIs. A range of possible reasons for unnecessary use of antibiotics by rural and township physicians have been identified. It is not uncommon for them to prescribe antibiotics ‘just in case’, due to a desire to reduce medical, legal and reputation risk in the face of diagnostic uncertainty [8]. Antibiotics may also be used unnecessarily under the driver of income interests, including prescribing of inappropriate antibiotics for particular diseases, unnecessary escalation (for example prescribing more expensive and broad-spectrum antibiotics when cheaper and more specific antibiotics can give the same result) and intravenous use [9, 10]. In addition, most rural and township physicians in China have to distinguish bacterial or virus infection based on patient-reported symptoms because microbiological facilities are not available at most front-line health care settings [11].

Most research on AMR in China has drawn on patient data collected in urban specialist hospitals. The often asserted high burden of AMR in China may be misleading since current assumptions are based on potential pathogens isolated in selected microbiology laboratories enrolled in the national surveillance system and it is uncertain if this reflects the incidence of resistance in non-hospitalized patients with mild to moderate infection receiving antibiotics, most of whom do not have samples sent to the laboratory [12, 13]. UK data indicates that 1:35 patients presenting to their general practitioners with acute cough have microbiological sampling; while the same sample proportion is only about 1:5 at secondary and tertiary medical care settings [14]. These data indicate that resistance rates to key antibiotics based on biased laboratory data may be over-emphasized [15–18]. Similarly, the often reported reduction in antibiotics prescription rate derived from paper or electronic medical records may also be misleading since such records in most primary clinics in China are incomplete or inaccurate and the bulk of antibiotics is used at primary care settings.

This paper tries to describe prevalence and patterns of antibiotics prescription and bacteria detection and sensitivity to antibiotics in rural China and implications for future antibiotic stewardship. It uses data from a mixed methods project co-sponsored by China National Natural Science Foundation and UK Research and Innovation [14].

## Methods

This study is part of the ‘pathways to optimizing antibiotic use in Anhui: identifying key determinants in community and clinical settings’ project. It used mixed-methods consisting of qualitative interviews, non-particative observations, semi-structured surveys and microbiological studies. The study protocol has been published separately [14]. This paper used mainly the quantitative data from the study.

### Setting

The study was implemented in one village clinic and one township health center in each of four rural residential areas in Anhui Province, China. Anhui Province is located in the middle east of China and has a population of 68.6 million of whom 57% live in rural areas. It has 968 hospitals, 1,941 community and 1,398 township health centers, and 15,288 village clinics.

### Participants

The study aimed to recruit 1,000 patients presenting with respiratory infection [14]. Inclusion criteria were male or female patients who were: a) 18 years or older and able to give consent to participate in the microbiological study and exit survey; b) presenting to the recruitment site for his/her current illness for the first time during the study period; and c) observed as having one or more of the following: exacerbation of chronic obstructive pulmonary disease (COPD), upper respiratory tract infection with productive cough or sore throat. Selection of these conditions was based on the consideration that there may be differences between patients with different clinical diagnosis (e.g., higher chances for identifying bacteria and antibiotics resistance among patients with exacerbation of COPD than those with cough and/or a sore throat). Acute otitis and rhinosinusitis were not included because these diseases happen mainly in children while this study population comprised only adults (over 18-year-old).

Patients were selected via “consecutive sampling” in which, when a start date had been determined, the recruitment continued daily (7 days a week) thereafter, between 8am-5 pm or 9am- 6 pm on alternate days, until the target numbers had been reached. All incoming patients to the site village clinics and township health centers who met the inclusion criteria during any study day were invited to participate. This was a pragmatic approach to sampling since patient record systems do not allow the flexibility to carry out random sampling, and ongoing recruitment ensured the most efficient use of staff and resources in this setting.

### Data collection

#### Semi-structured observations and exit surveys

A trained researcher was sent to each participating clinics and health centers to perform semi-structured observations and exist survey. The observation focused on daily operational routine including test ordering, prescribing, patient recall and other standard procedures using a pre-designed worksheet [14]. The exit survey used a questionnaire consisting of structured and semi-structured questions informed by open-ended interviews undertaken in the study’s pilot phase and included information on social demographics, symptoms and diseases history. The questionnaire was administered face-to-fact by the researcher for all patients consented by the attending clinicians at clinics and health centers and recruited into the study. The questionnaire was completed when the episode of care had concluded.

#### Specimen collection and microbiological testing

The study collected sputum and throat swabs for bacterial culture, identification and susceptibility testing. Sputum was collected from patients presenting with productive cough and throat swabs from patients with sore throat. Samples were collected by the attending doctor using a sterilized container and according to a standard protocol. The specimens were transported and tested at the Central Laboratory of Anhui Medical University (AMU). For details of the test procedures, please refer to our published protocol [14].

#### Data process and analysis

Recordings from the semi-structured observations were examined to see whether and which antibiotics were prescribed for the symptomatic RTI patients. Two researchers performed the examination independently and discordances were solved by discussions between them. For data from the semi-structured exit-survey, only the structured items were used in this study. This paper reports on descriptive analysis of: a) social demographics (sex, age and educations) of patients recruited; b) antibiotics use by clinical diagnosis; c) percentages of patients identified with specific types of bacteria by groups of clinical diagnosis; and d) prevalence rates of resistance of most frequently identified bacteria to commonly used antibiotics.

## Results

### Social demographics of informants

A total of 1073 patients meeting our inclusion criteria were invited to participate and 1068 (51% males and 49% females) provided specimens and completed the face-to-face survey. Of these, 683 provided sputum and 385 provided throat swab specimens. Over 60% of their visits to the clinics or health centers happened within 4 days after onset of infection symptoms and the mean time interval was 6 days. Over half (55%) of the patients had less than 5 years of education with 26% being illiterate. There were statistically significant differences between sex groups with males being older and more educated (Table 1).

**Table 1:** Socio-demographic characteristics of respondents

	Type of specimens		Total
	Sputum	Throat swab	
Age			
< = *39*	124 (18.2)	148 (38.4)	272 (25.5)
* 40–53*	168 (24.6)	114 (29.6)	282 (26.4)
* 54–64*	183 (26.8)	69 (17.9)	252 (23.6)
> = *65*	208 (30.5)	52 (13.5)	260 (24.3)
* Missing*	0 (0.0)	2 (0.5)	2 (0.2)
Education			
* 0*	211 (30.9)	65 (16.9)	276 (25.8)
* 1–5*	211 (30.9)	95 (24.7)	306 (28.7)
* 6–8*	121 (17.7)	108 (28.1)	229 (21.4)
> *8*	134 (19.6)	116 (30.1)	250 (23.4)
* Missing*	6 (0.9)	1 (0.3)	7 (0.7)
Sex			
* Male*	366 (53.6)	179 (46.5)	545 (51.0)
* Female*	317 (46.4)	206 (53.5)	523 (49.0)
Total	683 (64.0)	385 (36.0)	1068 (100.0)

### Clinical diagnosis and antibiotic use

Antibiotics were prescribed for 88% of all recruited patients and 36% of these prescriptions contained two or more types of antibiotics (Table 2). The most common clinical diagnosis were “respiratory tract infection”(31%), “bronchitis/tracheitis” (23%), “pharyngitis”(11%), “common cold”(8%), “pneumonia/bronchopneumonia” (5%) and “tonsillitis”(4%). Patients diagnosed with “bronchitis/tracheitis” witnessed the highest antibiotics prescription rate (94%), followed by “tonsillitis” (93), “pneumonia/bronchopneumonia” (91%) and “respiratory tract infection” (90%). Prescription of multiple (2 or more) antibiotics happened most frequently for COPD (76%), bronchitis/tracheitis (50%), tonsillitis (50%) and pneumonia/ bronchopneumonia (46%) patients. The most commonly used antibiotics were penicillins, cephalosporins and quinolones. In terms of the anatomical therapeutic chemical (ATC) classification, J01MA12 ranked the highest (31% in all the patients studied), followed by J01CA04 (15%), J01FA13 (15%), J01CR02 (15%) and J01DC02 (10%) (Additional file 1).

**Table 2:** Antibiotic use by clinical diagnosis, **N (%)**

	Patients	Any antibiotic	Number of antibiotics used			Category of antibiotics used			
			1	2	3 +	Quinolones	Cephalosporins	Penicillins	Others
**Diagnosis**									
* -Respiratory tract infection*	326 (30.5)	296 (90.8)	179 (54.9)	92 (28.2)	24 (7.4)	112 (34.4)	92 (28.2)	141 (43.3)	22 (6.7)
* -Bronchitis/tracheitis*	249 (23.3)	234 (94.0)	109 (43.8)	101 (40.6)	23 (9.2)	106 (42.6)	82 (32.9)	109 (43.8)	7 (2.8)
* -Pharyngitis*	119 (11.1)	104 (87.4)	84 (70.6)	15 (12.6)	4 (3.4)	17 (14.3)	20 (16.8)	54 (45.4)	10 (8.4)
* -Common cold*	85 (8.0)	69 (81.2)	57 (67.1)	7 (8.2)	5 (5.9)	12 (14.1)	50 (58.8)	11 (12.9)	5 (5.9)
* -Pneumonia/bronchopneumonia*	48 (4.5)	44 (91.7)	22 (45.8)	16 (33.3)	6 (12.5)	16 (33.3)	25 (52.1)	17 (35.4)	2 (4.2)
* -Tonsillitis*	44 (4.1)	41 (93.2)	19 (43.2)	15 (34.1)	7 (15.9)	18 (40.9)	17 (38.6)	18 (40.9)	1 (2.3)
* -COPD*	29 (2.7)	26 (89.7)	4 (13.8)	15 (51.7)	7 (24.1)	20 (69.0)	7 (24.1)	17 (58.6)	0 (0.0)
* -Others*	24 (2.2)	15 (62.5)	11 (45.8)	4 (16.7)	0 (0.0)	4 (16.7)	7 (29.2)	5 (20.8)	1 (4.2)
* -Diagnosis not given*	144 (13.5)	109 (75.7)	71 (49.3)	31 (21.5)	7 (4.9)	30 (20.8)	59 (41.0)	32 (22.2)	15 (10.4)
***P***		**0.000**	**0.000**	**0.000**	**0.002**	**0.000**	**0.000**	**0.000**	**0.015**
**Total**	1068 (100)	938 (87.8)	556 (52.1)	296 (27.7)	86 (8.1)	335 (31.4)	359 (33.6)	404 (37.8)	63 (5.9)

### Diagnosis and bacteria identification

Of all the specimens tested, 329 (31%) were isolated with pathogenic bacteria or asymptomatic carrier, and 23 of them, with two strains of bacteria. These 352 isolates comprised 85 (24%) for *K. pneumonia*, 57 (16%) for *H. influenzae*, 52(15%) for *H. parainfluenzae*, 22(6%) for *P. aeruginosa*, 18 (5%) for *S.aureus*, 12 (3%) for *M. catarrhalis*, 8 (2%) for *S. pneumoniae*, 6 (1%) for *β.haemolytic streptococci*, 5(1%) for *A.baumannii* and 4(1%) for *E.coli*. The study did not find statistically significant difference in the chances of bacteria isolation between patients with different clinical diagnosis. Table 3 provides bacterial detection results from sputum and throat swab specimens by diagnosis; while the results from sputum and throat swab specimens are given separately in Additional files 2 and 3.

**Table 3:** Bacterial detection by clinical diagnosis (*n* = 1086, sputum and throat swabs)

	Any bacteria	*K.pneumonia*	*H.influenzae*	*H.parainfluenzae*	*P.aeruginosa*	*S.aureus*	M.*catarrhalis*	S.*pneumoniae*	B.*haemolytic streptococci*	A.*baumannii*	E.*coli*
**Diagnosis**											
* -Respiratory tract infection*	92 (28.2)	32 (34.8)	12 (13.0)	19 (20.7)	3 (3.3)	6 (6.5)	1 (1.1)	2 (2.2)	2 (2.2)	2 (2.2)	1 (1.1)
* -Bronchitis/tracheitis*	83 (33.3)	21 (25.3)	19 (22.9)	8 (9.6)	7 (8.4)	5 (6.0)	6 (7.2)	3 (3.6)	1 (1.2)	0 (0.0)	1 (1.2)
* -Pharyngitis*	24 (20.2)	6 (25.0)	3 (12.5)	1 (4.2)	1 (4.2)	3 (12.5)	1 (4.2)	0 (0.0)	1 (4.2)	1 (4.2)	0 (0.0)
* -Common cold*	24 (28.2)	5 (20.8)	4 (16.7)	5 (20.8)	3 (12.5)	0 (0.0)	0 (0.0)	1 (4.2)	0 (0.0)	0 (0.0)	0 (0.0)
* -Pneumonia/bronchopneumonia*	20 (41.7)	5 (25.0)	8 (40.0)	2 (10.0)	1 (5.0)	0 (0.0)	2 (10.0)	0 (0.0)	0 (0.0)	0 (0.0)	0 (0.0)
* -Tonsillitis*	14 (31.8)	2 (14.3)	1 (7.1)	3 (21.4)	0 (0.0)	1 (7.1)	1 (7.1)	0 (0.0)	1 (7.1)	0 (0.0)	0 (0.0)
* -COPD*	11 (37.9)	1 (9.1)	2 (18.2)	2 (18.2)	2 (18.2)	0 (0.0)	0 (0.0)	1 (9.1)	0 (0.0)	0 (0.0)	1 (9.1)
* -Others*	7 (29.2)	1 (14.3)	0 (0.0)	2 (28.6)	1 (14.3)	0 (0.0)	0 (0.0)	1 (14.3)	0 (0.0)	1 (14.3)	0 (0.0)
* -Diagnosis not given*	54 (37.5)	12 (22.2)	8 (14.8)	10 (18.5)	4 (7.4)	3 (5.6)	1 (1.9)	0 (0.0)	1 (1.9)	1 (1.9)	1 (1.9)
*P*	0.064	0.471	0.103	0.384	0.454	0.625	0.292	0.278	0.825	0.180	0.540
**Total**	**329 (30.8)**	**85 (24.2)**	**57 (16.2)**	**52 (14.8)**	**22 (6.3)**	**18 (5.1)**	**12 (3.4)**	**8 (2.3)**	**6 (1.7)**	**5 (1.4)**	**4 (1.1)**

### Antibiotics prescription and bacteria sensitivity

**Fig. 1:**
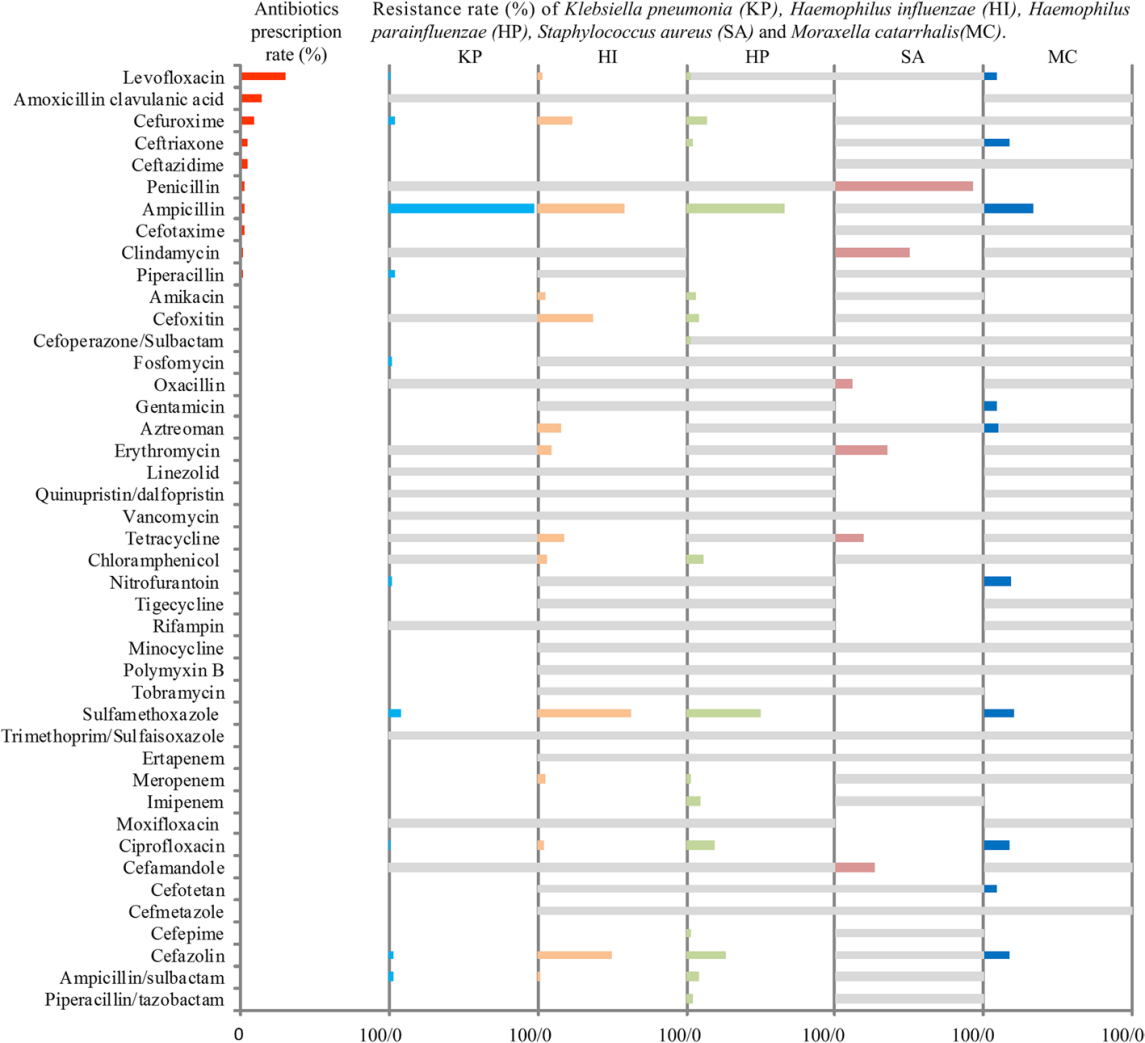
Antibiotics prescription rates compared with antibiotics resistance rates (grey bars represent not applicable or not performed tests)

Figure 1 portraits the prescription rate of specific antibiotics and resistance rate of the top 5 bacteria isolated from sputum and swab specimens. These bacteria were tested sensitive to most of the antibiotics studied. The highest resistance rate was estimated as 97.4% for K. *pneumoniae* to Ampicillin followed by 92.3 for S. *aureus* to Penicillin and 62.3% for H.*influenzae* to Sulfamethoxazole. No clear association between the frequency of antibiotics prescription and the rates of resistance was observable. The highest resistance rate to the five most frequently prescribed antibiotics was 30.0%.

## Discussion

### Key findings

This study reveals useful findings on antibiotic use and microbiological test results for patients presenting RTIs symptoms at rural and township care settings in Anhui, China. It documents an antibiotic prescription rate as high as 87.8% and most frequent use of broad-spectrum and multiple antibiotics. It demonstrates that nearly one third (30.8%) of the specimens were isolated with pathogenic bacteria or asymptomatic carrier with *the top bacteria strains being K. pneumonia, H. influenza, H. parainfluenzae, P. aeruginosa and S. aureus* and the highest resistance rate being *K. pneumoniae* to ampicillin followed by *S.pneumoniae* to Clindamycin, *H. influenzae* to Trimethoprim/ Sulfaisoxazole and *H. parainfluenzae* to Ampicillin.

### Implications in context of other research and for policy

The above study findings have important implications for antibiotics stewardship. The high rate of antibiotics prescriptions contradicts a common belief among policymakers in China that excessive antibiotics use is being brought under control as a result of the nationwide Special Antibiotics Use Rectification program (initiated in 2011) and the New Health System Reforms [19, 20]. These initiatives focus on antibiotics use at secondary and tertiary hospitals. Given that about 57% of China’s vast population lives in rural and township areas and over 70% of antibiotics prescriptions occur at settings in these areas [21, 22], there is a clear need for added attention on excessive antibiotics use at these settings and communities.

The frequent use of broad-spectrum and multiple (2 or more) antibiotics calls for stewardship programs aiming at not only reducing the number of prescriptions but also promoting single and narrow-spectrum antibiotics. Most of the broad-spectrum antibiotics, especially quinolones, cephalosporins and amoxi-clav, have strong resistance driving effect; while use of multiple antibiotics often acts as an even greater driver. This prevalent use of broad-spectrum and multiple antibiotics may be attributed mainly to medical uncertainty though decisions on which specific antibiotics to use depends on a variety of factors including availability, price, sensitivity, adverse effects and other characteristics of the antibiotics under concern [23–27]. Rural and township healthcare doctors in China work in a difficult situation in which microbiological tests are unavailable and it is hard to tell the pathogen and its sensitivity to specific antibiotics from clinical symptoms/ history. So, they tend to view broad-spectrum or combined antibiotics as a safer strategy than narrow spectrum antibiotics since the former have greater chance of hitting the actual pathogen [28, 29].

The microbiological test results indicate the feasibility of such tests for rural and township care attendees in resource-poor rural China. As described in our separate protocol paper, the testing proceeded by collecting specimens at the rural and township care settings and sending the specimens to a tertiary hospital with a microbiological lab via existing transportation services [14]. We collected and tested 1068 specimens out of 1073 RTI patients. Our overall rate of bacteria detection was 30.8% which is compatible with published results for similar population groups [30]. These all suggest that the testing is acceptable to both patients and physicians and the test results are relatively reliable.

The difference between the resistance rates tested in this study and that from higher level settings suggests a need for incorporating rural and township care settings into China’s national antibiotics use and resistance surveillance systems. Our study indicates that antibiotics resistance rates among rural and township care attendees are substantially lower than that among patients of hospitals forming the national antibiotics resistance surveillance network, being 2.5% vs 56.6% for K.*pneumonia* to cefazolin, 58.0% vs 60.3% for H.*influenzae* to ampicillin, 5.9% vs 25.2% for P.*aeruginosa* to piperacillin, 35.3% vs 61.5% for S.*aureus* to erythromycin and 80.0% vs 89.9% for S.*pneumoniae* to clindamycin [30]. These differences in resistance between hospitals and primary care would imply lower general antibiotics prescribing, and especially more narrow-spectrum antibiotics. However, as mentioned above, doctors at primary care settings are practicing the opposite.

The early healthcare seeking together with the high rate of antibiotics prescription highlights the importance of educating doctors and patients to postpone antibiotics prescription and use. The study found that over 60% of visits to the village clinics or health centers happened within 4 days after onset of infection symptoms and the mean time of visits was 6 days. This is relatively short as compared to that from western nations. Studies from Europe of lower RTIs have documented a mean time lag of 4 to 12 days [31]. Early visits to clinics with a clear expectation to get antibiotics merits adequate attention in future interventions.

Both the profiles of detectable pathogen and asymptomatic carrier bacteria have important implications. Detection of pathogen bacteria (e.g., S.*aureus*, M.*catarrhalis*, S.*pneumoniae*,*βhaemolytic streptococci* and E.*coli)* should inform clinical treatment decision-making. Although detection of the asymptomatic carrier may not necessarily cause the infections, it may be used as indirect or surrogate indicators for assessing antibiotic resistance. Perhaps the biggest barrier to microbiological evidence-based antibiotics use for primary care doctors at present may be the delay due to sending specimens to and getting results from higher level labs in addition to time needed for bacteria cultivation and test. Point of care tests may help overcome this barrier. Unfortunately, such tests are generally not available in China [32]. This lack of external validity and clinical uncertainty in primary care together with the links between antibiotics prescription and the number of days until care seeking call for more patient education and more use of a delayed prescribing strategy.

### Strengths and limitations of the study

This study has both strengths and limitations. It is the first study that collected data from healthcare providers and users via a non-participative observation whilst most of the existent research on antibiotics use in China uses data from medical records or reports by medical care givers who may be incentivized to omit recording overuse or misuse of antibiotics so as to meet relevant policy requirements. It is also the first study that performed both microbiological testing and clinical data collection at rural and township care settings and thus enables cross-linking between data from different sources. However, the study suffers from limited number of patients and site clinics. It involved only 8 site clinics or health centers from a single province. So, readers are cautioned about the generalization of the findings to other parts of China, thought the social, cultural and economic background of Anhui is similar to the majority of areas in the nation. The non-participative observation may also have intervened, to some extent, the routine encounters between the patients and doctors and the prescription behaviors being observed though we had arranged a two-week preparation for each site clinic to allow the field researchers to build trust with the doctors. The paper tried to use pure clinical diagnoses to evaluate the other findings; while in reality, it is generally hard to separate the diagnoses of respiratory tract infection and common cold, furthermore pharyngitis and tonsillitis.

## Conclusions

The study makes innovative use of observations in collecting data about antibiotics prescription at rural and township care settings in China for patients with respiratory tract infections and establishes the feasibility of conducting microbiological testing outside Tier 2 and 3 hospitals in rural China. It reveals that prescription of antibiotics, especially broad-spectrum and combined antibiotics, is still very common in rural China and there is a clear need for stewardship programs aimed at both reducing the number of prescriptions and promoting single and narrow-spectrum antibiotics. It also documents important differences in resistance between hospitals and primary care and calls for new specific guidelines for primary care based on findings from this and other papers and international expertise like that from the General Practice Respiratory Infections Network.

## Supplementary Information

**Additional file 1.**

**Additional file 2.**

**Additional file 3.**

## Data Availability

Data are available from the University of Bristol or Anhui Medical University Data Access (contact via H.Lambert@bristol.ac.uk/dbwang@vip.sina.com) for researchers who meet the criteria for access to confidential data.

## Abbreviations

AMR: Antimicrobial resistance
COPD: Chronic obstructive pulmonary disease
RTIs: Respiratory tract infections
AMU: Anhui Medical University
OR: Odds ratio
