# Supplementary material for: Clinical diagnosis and treatment of common respiratory tract infections in relation to microbiological profiles in rural health facilities in China: implications for antibiotic stewardship
Source: BMC Fam Pract. 2021 May 6;22:87. doi: 10.1186/s12875-021-01448-2 (PMC8103749; doi:10.1186/s12875-021-01448-2)
Supplement: Supplementary file 1 — Additional file 1. [file 12875_2021_1448_MOESM1_ESM.docx]

Additional file 1 Most frequently used antibiotics by clinical diagnosis

| **Symptom** | **ATC Codes** | | | | | | | | | |
| --- | --- | --- | --- | --- | --- | --- | --- | --- | --- | --- |
|  | **J01MA12** | **J01CA04** | **J01FA13** | **J01CR02** | **J01DC02** | **J01DB09** | **J01DD04** | **J01DD02** | **J01CE01** | **J01CA01** |
| **Diagnosis** |  |  |  |  |  |  |  |  |  |  |
| *-Bronchitis/tracheitis* | 106(42.6) | 31(12.4) | 66(26.5) | 61(24.5) | 24(9.6) | 9(3.6) | 11(4.4) | 13(5.2) | 7(2.8) | 6(2.4) |
| *-RTIs* | 110(33.7) | 66(20.2) | 39(12.0) | 47(14.4) | 33(10.1) | 13(4.0) | 24(7.4) | 8(2.5) | 22(6.7) | 15(4.6) |
| *-Pharyngitis* | 16(13.4) | 33(27.7) | 22(18.5) | 17(14.3) | 3(2.5) | 4(3.4) | 3(2.5) | 0(0.0) | 3(2.5) | 0(0.0) |
| *-Common cold* | 12(14.1) | 3(3.5) | 6(7.1) | 8(9.4) | 6(7.1) | 32(37.6) | 4(4.7) | 3(3.5) | 0(0.0) | 0(0.0) |
| *-Pneumonia/bronchopneumonia* | 16(33.3) | 4(8.3) | 6(12.5) | 8(16.7) | 2(4.2) | 2(4.2) | 6(12.5) | 10(20.8) | 0(0.0) | 0(0.0) |
| *-Tonsillitis* | 18(40.9) | 4(9.1) | 9(20.5) | 4(9.1) | 6(13.6) | 0(0.0) | 1(2.3) | 7(15.9) | 3(6.8) | 2(4.5) |
| *-COPD* | 20(69.0) | 1(3.4) | 9(31.0) | 10(34.5) | 2(6.9) | 0(0.0) | 3(10.3) | 0(0.0) | 2(6.9) | 1(3.4) |
| *-Others* | 4(16.7) | 3(12.5) | 2(8.3) | 1(4.2) | 3(12.5) | 0(0.0) | 0(0.0) | 1(4.2) | 1(4.2) | 0(0.0) |
| *-Not given* | 29(20.1) | 17(11.8) | 2(1.4) | 2(1.4) | 26(18.1) | 8(5.6) | 6(4.2) | 12(8.3) | 12(8.3) | 12(8.3) |
| P | 0.000 | 0.000 | 0.000 | 0.000 | 0.005 | 0.000 | 0.092 | 0.000 | 0.022 | 0.003 |
| **Total** | 331(31.0) | 162(15.2) | 161(15.1) | 158(14.8) | 105(9.8) | 68(6.4) | 58(5.4) | 54(5.1) | 50(4.7) | 36(3.4) |
