# Supplementary material for: Clinical diagnosis and treatment of common respiratory tract infections in relation to microbiological profiles in rural health facilities in China: implications for antibiotic stewardship
Source: BMC Fam Pract. 2021 May 6;22:87. doi: 10.1186/s12875-021-01448-2 (PMC8103749; doi:10.1186/s12875-021-01448-2)
Supplement: Supplementary file 2 — Additional file 2. [file 12875_2021_1448_MOESM2_ESM.docx]

Additional file 2 Bacteria isolated from sputum samples by clinical diagnosis (n=683)

| **Symptom** | Any bacteria | *K.pneumonia* | *H.influenzae* | *H.parainfluenzae* | *P.aeruginosa* | *S.aureus* | M.*catarrhalis* | S.*pneumoniae* | A.*baumannii* | E.*coli* | B.*haemolytic streptococci* |
| --- | --- | --- | --- | --- | --- | --- | --- | --- | --- | --- | --- |
| **Diagnosis** | | | | | | | | | | | |
| *-Bronchitis/tracheitis* | 72(36.2) | 18(9.0) | 17(8.5) | 7(3.5) | 6(3.0) | 4(2.0) | 6(3.0) | 3(1.5) | 0(0.0) | 0(0.0) | 1(0.5) |
| *-RTI* | 71(38.2) | 26(14.0) | 10(5.4) | 10(5.4) | 3(1.6) | 4(2.2) | 1(0.5) | 2(1.1) | 2(1.1) | 1(0.5) | 0(0.0) |
| *-Pharyngitis* | 11(29.7) | 3(8.1) | 3(8.1) | 0(0.0) | 0(0.0) | 1(2.7) | 1(2.7) | 0(0.0) | 1(2.7) | 0(0.0) | 0(0.0) |
| *-Common cold* | 18(25.7) | 5(7.1) | 4(5.7) | 1(1.4) | 3(4.3) | 0(0.0) | 0(0.0) | 1(1.4) | 0(0.0) | 0(0.0) | 0(0.0) |
| *-Pneumonia/bronchopneumonia* | 18(40.9) | 5(11.4) | 8(18.2) | 1(2.3) | 1(2.3) | 0(0.0) | 1(2.3) | 0(0.0) | 0(0.0) | 0(0.0) | 0(0.0) |
| *-Tonsillitis* | 3(37.5) | 1(12.5) | 1(12.5) | 1(12.5) | 0(0.0) | 0(0.0) | 0(0.0) | 0(0.0) | 0(0.0) | 0(0.0) | 0(0.0) |
| *-COPD* | 11(39.3) | 1(3.6) | 2(7.1) | 2(7.1) | 2(7.1) | 0(0.0) | 0(0.0) | 1(3.6) | 0(0.0) | 1(3.6) | 0(0.0) |
| *-Others* | 7(38.9) | 1(5.6) | 0(0.0) | 2(11.1) | 1(5.6) | 0(0.0) | 0(0.0) | 1(5.6) | 1(5.6) | 0(0.0) | 0(0.0) |
| *-Not given diagnosis* | 41(44.1) | 11(11.8) | 7(7.5) | 4(4.3) | 4(4.3) | 2(2.2) | 1(1.1) | 0(0.0) | 1(1.1) | 1(1.1) | 0(0.0) |
| P | 0.513 | 0.637 | 0.220 | 0.384 | 0.668 | 0.880 | 0.541 | 0.560 | 0.215 | 0.356 | 0.965 |
| **Total** | **252(36.9)** | **71(10.40)** | **52(7.61)** | **28(4.11)** | **20(2.9)** | **11(1.61)** | **10(1.46)** | **8(1.17)** | **5(0.7)** | **3(0.4)** | **1(0.1)** |
