# Supplementary material for: Clinical diagnosis and treatment of common respiratory tract infections in relation to microbiological profiles in rural health facilities in China: implications for antibiotic stewardship
Source: BMC Fam Pract. 2021 May 6;22:87. doi: 10.1186/s12875-021-01448-2 (PMC8103749; doi:10.1186/s12875-021-01448-2)
Supplement: Supplementary file 3 — Additional file 3. [file 12875_2021_1448_MOESM3_ESM.docx]

Additional file 3 Bacteria isolated from throat swabs by clinical diagnosis(n= 385)

| **Symptom** | Any bacteria | *K.pneumonia* | *H.influenzae* | *H.parainfluenzae* | *P.aeruginosa* | *S.aureus* | M.*catarrhalis* | E.*coli* | B.*haemolytic streptococci* |
| --- | --- | --- | --- | --- | --- | --- | --- | --- | --- |
| **Diagnosis** |  |  |  |  |  |  |  |  |  |
| *-Bronchitis/tracheitis* | 11(22.0) | 3(6.0) | 2(4.0) | 1(2.0) | 1(2.0) | 1(2.0) | 0(0.0) | 1(2.0) | 0(0.0) |
| *-RTI* | 21(15.0) | 6(4.3) | 2(1.4) | 9(6.4) | 0(0.0) | 2(1.4) | 0(0.0) | 0(0.0) | 2(1.4) |
| *-Pharyngitis* | 13(15.9) | 3(3.7) | 0(0.0) | 1(1.2) | 1(1.2) | 2(2.4) | 0(0.0) | 0(0.0) | 1(1.2) |
| *-Common cold* | 6(40.0) | 0(0.0) | 0(0.0) | 4(26.7) | 0(0.0) | 0(0.0) | 0(0.0) | 0(0.0) | 0(0.0) |
| *-Pneumonia/bronchopneumonia* | 2(50.0) | 0(0.0) | 0(0.0) | 1(25.0) | 0(0.0) | 0(0.0) | 1(25.0) | 0(0.0) | 0(0.0) |
| *-Tonsillitis* | 11(30.6) | 1(2.8) | 0(0.0) | 2(5.6) | 0(0.0) | 1(2.8) | 1(2.8) | 0(0.0) | 1(2.8) |
| *-COPD* | 0(0.0) | 0(0.0) | 0(0.0) | 0(0.0) | 0(0.0) | 0(0.0) | 0(0.0) | 0(0.0) | 0(0.0) |
| *-Others* | 0(0.0) | 0(0.0) | 0(0.0) | 0(0.0) | 0(0.0) | 0(0.0) | 0(0.0) | 0(0.0) | 0(0.0) |
| *-Not given diagnosis* | 13(25.5) | 1(2.0) | 1(2.0) | 6(11.8) | 0(0.0) | 1(2.0) | 0(0.0) | 0(0.0) | 1(2.0) |
| P | 0.072 | 0.965 | 0.765 | 0.006 | 0.837 | 0.928 | 0.053 | 0.567 | 0.986 |
| **Total** | **77(20.0)** | **14(3.6)** | **5(1.3)** | **24(6.2)** | **2(0.8)** | **7(1.8)** | **2(0.5)** | **1(0.3)** | **5(1.3)** |
